# TSProm: deep learning framework to predict tissue-specific regulatory logic

**DOI:** 10.1093/nargab/lqag050

**Published:** 2026-06-03

**Authors:** Pallavi Surana, Pratik Dutta, Nimisha Papineni, Rekha Sathian, Zhihan Zhou, Han Liu, Ramana V Davuluri

**Affiliations:** Department of Biomedical Informatics, Stony Brook University, NY 11794, United States; Department of Biomedical Informatics, Stony Brook University, NY 11794, United States; Department of Biomedical Informatics, Stony Brook University, NY 11794, United States; Department of Biomedical Informatics, Stony Brook University, NY 11794, United States; Department of Computer Science, Northwestern University, IL 60208, United States; Department of Computer Science, Northwestern University, IL 60208, United States; Department of Biomedical Informatics, Stony Brook University, NY 11794, United States

## Abstract

Characterizing tissue-specific (TSp) gene expression is crucial for understanding development and disease; however, traditional expression-based methods often overlook the latent “regulatory grammar” embedded in non-coding DNA, particularly across distal promoter regions. Here, we introduce *TSProm*, a framework that adapts a DNA foundation model (DNABERT2) to decode the regulatory logic of TSp promoters at the isoform level. Our contributions are two-fold: (i) a comparative design that trains two specialized models: Model A for general promoter biology and Model B for tissue-specific regulation enabling precise isolation of sequence motifs surrounding the transcription start site that uniquely define tissue identity; (ii) we develop an explainable AI module that integrates attention-based motif discovery with model-agnostic SHAP analysis to yield cross-validated interpretations of learned features. Applying TSProm to human brain, liver, and testis promoters, we identified clinically relevant transcription factors (TFs) in the brain, including *SP1, MYC*, and *HES6*, whose associations with gliomas and neuroblastomas highlight clinical relevance. Moreover, our results highlight *C2H2* zinc finger proteins as a dominant family shaping the global landscape of TSp gene regulation. TSProm provides an interpretable and generalizable framework for identifying tissue-specific regulatory elements, offering powerful computational tools to investigate gene regulation in both normal and disease contexts.

## Graphical Abstract

**Graphical Abstract:**
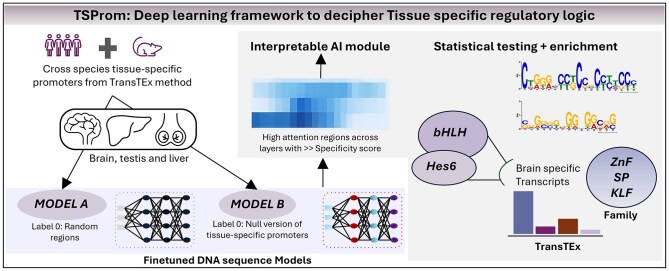

## Introduction

Precise regulation of gene expression underlies development, homeostasis, and disease in multicellular organisms. Distinct tissues and cell types exhibit unique transcriptional programs shaped by chromatin state, transcription factor (TF) binding, and sequence context [1, 2]. Disruption of these regulatory mechanisms contributes to conditions ranging from cancer to neurodegeneration and often presents in tissue/cell-type specific manner [3]. Despite extensive transcriptomic profiling, the sequence features encoding tissue-specific (TSp) regulation remain incompletely understood. Regulatory elements controlling TSp promoter usage play an essential role in organ development and disease dysregulation, with widespread regulation of alternative messenger RNA (mRNA) transcripts further influencing promoter activity [1, 4, 5]. mRNA transcripts are molecules thay carrry genetic information frrom DNA to ribosomes for protein synthesis. Understanding their language, taking long-range context is essential for revealing which functional modules get disrupted in complex diseases such as cancer, where promoters often lose their tissue specificity and acquire increased plasticity [2, 6, 7].

Traditional approaches to characterize tissue specificity focus on differential expression or marker genes, which overlook the latent “regulatory grammar” embedded in non-coding promoter and enhancer sequences [2, 8]. In addition, most methods focus on core and proximal promoters [regions upstream of the transcription start sites (TSS)] and not distal regions. Recent advances in transformer architectures have shown that deep learning models can extract biologically meaningful representations directly from raw DNA across both coding and non-coding genomic regions like promoters, enhancers, silencers, insulators, splice sites, and more [9–16].

Utilizing the capability of these models and their significant applications in understanding alternative promoters’ regulatory regions, we introduce a gene isoform-level Tissue Specific Promotor DNA Language model *TSProm*, extending DNABERT2, a second-generation transformer-based genomic sequence-only foundation model to classify and decipher TSp promoter biology. It utilizes Byte Pair Encoding (BPE) tokenization and Attention with Linear Biases (ALiBi) to efficiently process long, multi-species DNA sequences [9, 11]. We wanted to explore tissue specificity in normal tissues so that we can develop an understanding of the specific rules of normal tissues and further validate their relevance in complex genomic diseases.

We implemented TransTEx [4] to curate promoter expression groups for Mouse BodyMap dataset [17] to generate Mouse expression groupings and access human expression groups available at TransTExdb. Promoters were classified into five groups: TSp, representing transcripts uniquely expressed in a single tissue; TEn, transcripts expressed in one or more but fewer than 50% of tissues (where total is 26 major tissues from GTEx expression dataset); Wide, transcripts expressed in >50% of tissues; Low, transcripts with low expression or limited to a small subset of samples across all tissues; and Null, transcripts with no detectable expression or expressed only in a minimal number of samples across tissues. We extended regions around the TSS for the promoters grouped by tissue to capture both proximal and distal promoter interactions. For both human and mouse, we focused on three representative tissues based on the number of TSp transcripts: testis, liver, and brain for fine-tuning DNABERT2 and compared their performance with GENA-LM and Nucleotide Transformer model [11, 13, 14].

To interpret predictions, we implemented a comprehensive attention-guided motif (short repeats with biological meaning) discovery module, cross-validated [12] with SHapley Additive exPlanations (SHAP)-based scores from game theory [18], which discovers and verifies sequence motifs most relevant to tissue specificity. This explainable AI (xAI) approach improves the understanding of TFs in the distal promoter and regions beyond, across tissues. The module also reveals candidate regulatory motifs consistent with known TF binding patterns. We further validate the findings with publicly available experimentally validated datasets in literature like JASPAR 2024 CORE database [19], TF-Marker [20], and KnockTF 2.0 [21]. In addition, we validated significant motifs with the ENCODE ChIP-seq peaks [22] enriched to strengthen these findings.

TSProm demonstrates that transformer-based encoder DNA Foundation Models can be effectively specialized to capture long-range TSp promoter logic, providing high predictive performance. The xAI module gives insights into the tokens/motifs (DNA language words) that are potentially responsible for tissue specificity of isoform-level gene regulation. This understanding helps in the identification of relevant TFs and the target genes that are dysregulated in complex multifactorial genomic diseases.

## DNA foundation models

DFMs are deep learning models trained with self-supervised learning on large unlabeled datasets such as genomes. By enabling attention outputs, they provide detailed maps that offer xAI into how nucleotide tokens contribute across transformer layers [12, 23]. To evaluate the specific advantages of leveraging large-scale DFMs, we compare against recent deep learning architectures like IChrom-Deep-focal (sequence only model), with comparable performance to a transformer-based architecture and iPro-WAEL, which is an ensemble architecture between random forest and CNN architecture to identify promoters [16, 24].

We employed three state-of-the-art DFMs for sequence analysis: DNABERT-2, Nucleotide Transformer, and Gena-LM. DNABERT-2, a bidirectional transformer encoder, was utilized for its superior performance in representation learning tasks. The model processes DNA sequences BPE tokenization to replace the kmer tokenization to generate contextual embeddings through masked language modeling pre-training on multi-species genomic data. The model has 12 transformer encoder layers, 12 self-attention heads, hidden dimension of 768, and approximately 117 million parameters. This also aligns with the semantics of DNA sequences, where information context naturally varies with sequence length. We fine-tuned DNABERT-2 using task-specific labeled datasets with a learning rate of (2e-5, 1e-5, 3e-4, 5e-6, and others) and batch size of 32 or 64 [11, 25].

Gena-LM employs an autoregressive transformer decoder architecture with 24 layers, 1024 hidden dimensions, and 16 attention heads, and was selected for superior performance in sequence understanding and attention interpretability. This model uses single nucleotide tokenization and next-token prediction objectives during pre-training [14, 25].

The Nucleotide Transformer (NT 500M–1000G) model is a bidirectional transformer encoder based on the BERT architecture, comprising approximately 500 million parameters. It is pre-trained using a masked language modeling objective on a diverse corpus of 3200 human genomes and 850 genomes from other species, enabling cross-species representation learning and contextual understanding of genomic sequences. Unlike previous models, NT replaces overlapping with non-overlapping *k*-mer tokenization [13, 25].

All three DFMs were implemented using PyTorch and fine-tuned on NVIDIA A40 GPUs with gradient accumulation steps of 1 to accommodate memory constraints.

## Datasets

We prepared multiple datasets to identify which combination captures the TSp regulation. The positive class is the TSp group (brain, testis, and liver). We chose these three tissues as they had the highest number of TSp transcripts across human and mouse common organs. (Appendix A: Supplementary Table S1). Negative class is multiple models using Wide, Null, TEn, and Low for benchmarking. We will explain the negative sets for Models A and B in the Materials and methods section.

We use the tissue–transcript groupings from TransTExdb (accessed December 2, 2024) and ran TransTEx on Mouse BodyMap data from [17] to generate Mouse TransTEx. We then combine the TSp groups from both human and mouse to generate a cross-species TSp dataset [4]. We construct a train, validation, and test split (80:10:10) of the top three TSp sites (testis, brain, and liver) together with the four-remaining expression-based groups for fine-tuning to control class imbalance. This setup was evaluated with DNABERT2, NT, and GENA-LM DNA Foundation Models (DFMs).

### Varying sequence lengths tested

We utilized transcriptomic groupings for human promoters from both the Mouse (mm39) and human (hg38) genomes. For these species, TSp promoters for the top three tissues (label 1), while promoters of the other group (label 0). We evaluated three promoter window configurations: −3 kb to +1 kb, −2 kb to +1 kb, and −1 kb to +1 kb relative to the TSS, to test core, proximal, and distal promoter context on model performance. This ensures we identify additional DNA tokens/motifs that localize near TSS apart from the core and proximal promoters [26, 27]. To determine biologically relevant sequence lengths, we analyzed the TFBS and histone mark profiles from ENCODE and studied the enrichment of peaks around TSS as summarized in (Appendix B: Supplementary Fig. S1). We found enrichment in regions 2k bp upstream and 1k bp downstream for most promoter related transcription factor-binding site (TFBS). This allows the models we built to capture core, proximal distal regulatory signals related to tissue specificity.

### Tackling data redundancy

To reduce redundancy among the sequences, we applied MMseqs2 clustering using “mmseqs easy cluster” with a minimum sequence identity and coverage threshold of 80% [28]. This is done to retain only representative sequences from each cluster, yielding a non-redundant set for fine-tuning. This is a key strategy to mitigate overfitting along with evaluating multiple promoter window sizes and validating performance across independent validation and held-out test sets. These are benchmarked across three DFMs and two traditional deep learning methods and the best one in terms of sequence lengths and performance metrics are chosen.

## Materials and methods

### Fine tuning of DFMs

**Figure 1.**
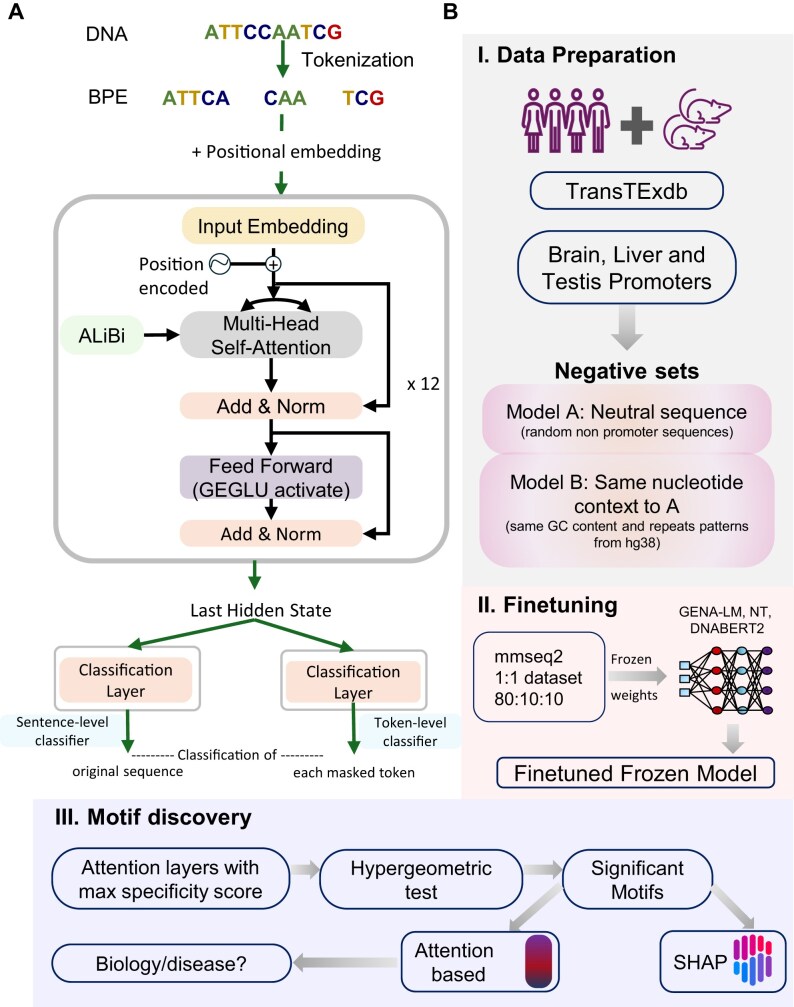
Overview of the TSProm framework. (**A**) Schematic of DNABERT2 architecture. (**B**) (I) Data preparation from multiple species and sources, including two negative sets used to construct Models A and B, respectively. (II) Fine-tuning using pre-trained genomic large language models (DNABERT2, Nucleotide Transformer, and GENA-LM). (III) Explainable AI and motif discovery workflow depicting attention-based interpretation and motif enrichment analysis.

To classify TSp groups from other expression categories, we fine-tuned DFMs using LoRA to minimize memory and compute requirements from HuggingFace. GENA-LMs’s AIRI-Institute/gena-lm-bigbird-base-t2t model variant utilizes BigBird-based sparse attention and ALiBi positional encoding to handle long DNA sequences efficiently, whereas DNABERT2’s zhihan1996/DNABERT-2–117M model handles a range of short to longer lengths with improved speed. DNABERT-2 tokenizes DNA sequences using BPE and embeds them into a high-dimensional space, which is then processed by 12 Transformer encoder layers. These layers incorporate ALiBi for distance-aware attention and GEGLU-activated feed-forward networks, producing a 768-dimensional output embedding for downstream analysis (Fig. 1A). These models have shown superior performance in longer sequence ranges for promoters [11, 25]. We finetune with Nucleotide Transformer’s InstaDeepAI/nucleotide-transformer-500m-1000g pre-trained on whole genome DNA sequences over 3000 diverse genomes [13].

### Models A and B of promoter biology

To understand the language of TSp gene regulation, we created negative set against a set of human and mouse promoters with sequence lengths of upstream (1k, 2k, and 3k) and downstream of 1k bp (5′–3′). We used these sequence lengths because of their distribution around TSS sites in TF binding regions, which are critical for promoter regulation (Appendix B). Model B: Negative set of this binary classification was same GC content and repeats patterns from the genome [29] and Model A: where we randomly selected genomic locations outside promoter sequences, ensuring that negative and positive samples do not overlap [13, 25] (Fig. 1B: Modules I and II). The data split of positive and negative class was 1:1 with test:train:dev in the ratio 80:10:10.

### Model evaluation metrics

We evaluated our fine-tuned models using key metrics: Accuracy, F1-Score, MCC, Precision, and Recall. Accuracy measures the rate of correct predictions, F1-score assesses precision and recall balance, and MCC provides a robust correlation coefficient regardless of class size evaluates the model’s ability to distinguish between classes at various thresholds. These metrics collectively ensure a thorough assessment of precision, recall, classification quality, and discriminative capacity (Appendix C).

### Attention-based motif discovery

#### Specificity scoring per layer and motif selection

We used the best fine-tuned models to extract attention-based motifs and assess their enrichment relative to the TSS (Fig. 1: Module III). For each sequence, token-level attention scores were obtained by averaging across all attention heads within each layer and then normalized relative to the maximum score per sequence.

Let q* denote the query vector corresponding to the special classification token [CLS], which is added to the beginning of each input sequence and used for downstream prediction representing a given sequence. Let *d* be the hidden dimension of q*, and let *k*_*k*_represents the key vector of the *j*-th BPE token, where *j* ∈ {1, …, J}, and *J* is the total number of tokens in the sequence.

The attention weight assigned to token *j* across all *H* attention heads is defined as:


(1) \begin{eqnarray*}
{\alpha _j} = \,\,\mathop \sum \limits_{h = 1}^H \frac{{exp\left( {\frac{{{q^T}{k_j}}}{{\sqrt d }}} \right)}}{{\mathop \sum \nolimits_{l = 1}^J exp\left( {\frac{{{q^T}{k_l}}}{{\sqrt d }}} \right)}}\,\,
\end{eqnarray*}

Here, ${\alpha _j}$ quantifies how strongly the global [CLS] representation attends to token *j*, and thus provides an attention score for sequence segments of variable length [11, 12].

Normalized token-level attention scores are defined:


(2) \begin{eqnarray*}
{\hat s_j}\,\, = \,\,\frac{{{\alpha _j}\,\,}}{{\max \left( \alpha \right)}}
\end{eqnarray*}

where max(α*_j_*) is the maximum attention weight within the sequence.

#### Layer specificity

To identify the most informative layers, we defined a specificity score based on the idea of *z*-score:


(3) \begin{eqnarray*}
Sp\,\, = \,\,\frac{{\max \left( {{{\hat s}_j}} \right) - \mu }}{\sigma }
\end{eqnarray*}

where $\hat s$ are the normalized attention scores, and μ, σ denote their mean and standard deviation, respectively. Based on this metric, the average of the middle layers (5, 6, and 7) together with the final layer were identified as the most informative across models, which were selected for motif discovery.

#### Motif selection

Tokens were considered candidate motifs if they satisfied:


(4) \begin{eqnarray*}
{\hat s_j}\,\, > \,\,\mu ,{\hat s_j} > \,\,2*\min \left( {\hat s} \right),\,\,\textit{length} \ge \,\,4bp
\end{eqnarray*}

Enrichment in positive versus negative sequences was tested using the hypergeometric distribution:


(5) \begin{eqnarray*}X & \sim &\textit{Hypergeom}( {N,K,n}),P( {X = k} ) = \frac{{\left( {\begin{array}{@{}*{1}{c}@{}} K\\ k \end{array}} \right)\left( {\begin{array}{@{}*{1}{c}@{}} {N - K}\\ {n - k} \end{array}} \right)}}{{\left( {\begin{array}{@{}*{1}{c}@{}} N\\ n \end{array}} \right)}}\end{eqnarray*}

where *N* is the total number of sequences, *K* the number of positive sequences, *n* the number of motif-containing sequences, and *k* the number of positives containing the motif [12, 30]. This distribution models the probability of a specific success in a sample drawn without replacement from a finite population set.

Ahocorasick algorithm was applied to [12, 31] for multi-pattern matching and ensures that motifs are present at least once. False discovery rate (FDR) correction with $\alpha \,\, = \,\,0.01$ was applied to identify significantly enriched motifs. Motifs with fewer than three instances were discarded.

Redundant motifs were merged using gapless overlap, and $ \pm 12$ base pair (bp) sequence windows around motif centers were extracted for visualization. *De novo* motif discovery was performed with MEME, and identified motifs were compared against the JASPAR 2024 CORE database using TOMTOM for TF annotation [19, 32].

#### Validation of motifs: SHAP analysis

SHAP analysis leverages cooperative game theory to quantify the biological importance of sequence motifs by calculating their average marginal contribution across every possible combination of TSp sequences, ensuring a model agnostic, fair, and consistent attribution for each sequence in its contribution to the TSp regulatory logic. We applied SHAP KernelExplainer to interpret finetuned DNABERT2 predictions at the token level [18, 33]. Each promoter sequence was tokenized, and a background baseline was constructed by replacing all tokens with the model’s mask token. Using this baseline, KernelExplainer estimated SHAP values for the positive class, quantifying each token’s contribution to the classification logits [34]. To interpret the contribution of sequence tokens, we computed SHAP values for each token across all input sequences.

For every unique token, we calculated the mean SHAP value, its absolute mean SHAP value, the frequency of occurrence, and the standard deviation. A 95% confidence interval (CI) for the mean was estimated as $1.96\,\,x\,\,\frac{\sigma }{{\sqrt n }}$, where $\sigma $ is the standard deviation of SHAP values and *n* is the number of token occurrences. Tokens with less occurrences were excluded to avoid noise. Tokens were then ranked either by frequency, mean SHAP value, or absolute mean SHAP value, and the top *k* tokens were selected for visualization. Relative importance of the top positive and negative tokens mean SHAP values were plotted (Fig. 3D).

#### Motif positional analysis

Consensus motif positions were identified by taking similar motif tokens and merging them using a fast gap-free alignment heuristic. Here, motifs were greedily collapsed if their best ungapped overlap exceeded a minimum similarity threshold based on the length of the shorter motif. The genomic start coordinate of each motif was then computed by adjusting the sequence start or end depending on the strand orientation. Motif-TSS distances were obtained as the difference between the motif start site and TSS. To visualize positional preferences, motif-TSS distances were aggregated into 100 bp bins, and the mean attention score was calculated per bin. A three-bin rolling average (∼300 bp smoothing) was applied to reduce local variability. Distributions were visualized using kernel density estimation (KDE) alongside histograms, with the TSS marked at 0 on the *x*-axis.

### Clinical and biological relevance of motifs

Predicted motif regions in TSp promoter sequences were validated by cross-referencing the corresponding TFs with TF-Marker [20] and KnockTF 2.0 [21] databases. TF-Marker provides experimentally verified TFs that act as cell type- or cell line-specific markers, many of which are critical for cancer cell identity and tumor heterogeneity. KnockTF 2.0 compiles transcriptomic profiles following TF perturbations, offering insights into regulatory pathways and oncogenic mechanisms controlled by them. Together, these resources enabled us to confirm both the TSp and cancer-relevant roles of the significant TFs.

To establish the significance of motif finding using traditional methods we use the sequences of 3k bp length and test the motifs for brain. We compare the similar motifs we find and summarize with Venn diagrams by running XSTREME tool and reporting JASPAR2026_CORE_vertebrates_non redundant database motifs [19, 35]. To further validate the TSp regulatory logic of identified regions by TSProm, we utilized the ENCODE dataset, comprising ∼4000 ChIP-seq files across ∼700 experiments for TF-binding sites [36]. We overlapped significant motif regions identified by TSProm (specifically from Model B) with aggregated human cCREs. We find significant peaks based on proportions of the background peaks found and rank them as suggested in other motif and peak scoring methods [37, 38].

## Results

### TSp promoter prediction models

The TSProm model is built upon the multi-genome pre-trained DFM, DNABERT2. To adapt the pre-trained model parameters for TSp classification and interpretability of the significant tokens/motifs, we employed transfer learning techniques by using the frozen weights from the model. The dataset was prepared by implementing the TransTEx method using data from the TransTExdb database, which divides the transcriptome into five expression groups based on isoform-level expression across normal tissues [4]. We applied this methodology to Mouse BodyMap expression data [17] to construct the MouseTransTEx dataset.

**Figure 2.**
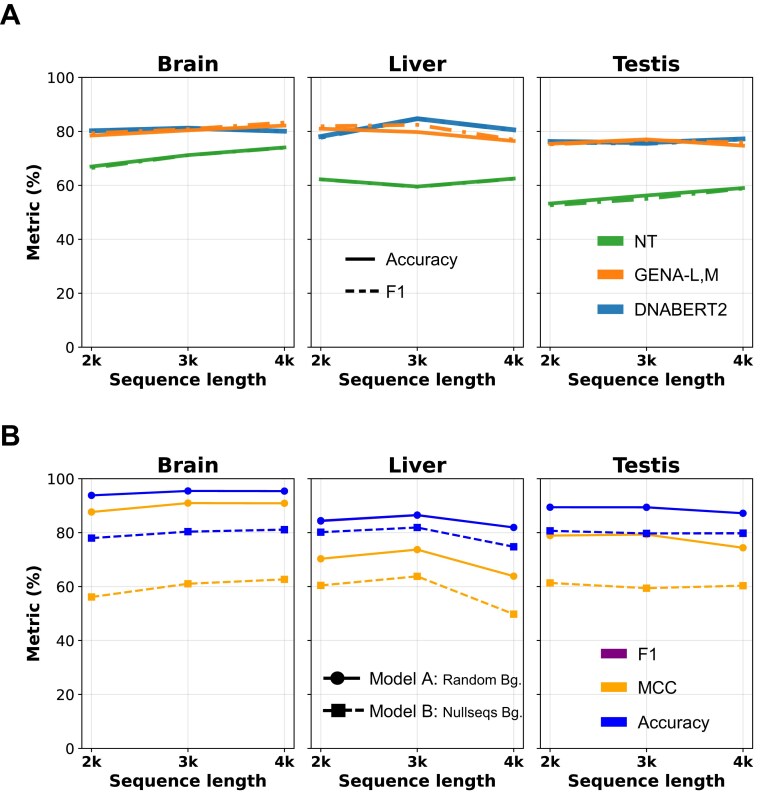
**A**) Fine-tuning performance of DNABERT2 (blue), NT (500m–1000G) (green) and GENA-LM (orange) on TSp (colored lines) versus other expression groups across the top three tissues, showing F1 (- - -) and accuracy(—). (**B**) Comparative performance of Models A and B for DNABERT on TSp data. The dashed lines represent (-□-) Model B: Nullseqs Bg.

We wanted to benchmark against recent attention-based DFM models; so, we chose GENA-LM, NT, and DNABERT2. We observed similar performances on fine-tuning tasks across 2k, 3k, and 4k lengths around the TSS between DNABERT2 and GENA-LM. NT (500m–1000G) performs the least accurate in this task (Fig. 2A). In fact, DNABERT2 models show slightly higher accuracy and F1 scores. Hyperparameter tuning was performed based on the learning rate, batch size, number of epochs, weight decay, and dropout rate. We also used a Nucleotide transformer model to benchmark summarized in Appendix D, Supplementary Table S2. We train models for each of the top three TSp sites (testis, liver, and brain) versus Null, Low, Wide, and TEn groups, and plot the best result from each tissue (Fig. 2A). An important factor underlying the limited performance of our models may be the choice of sequence window (∼80% F1). Regions 1 kb downstream of the TSS include the first intron of regulatory promoters, which is underexplored due to its indirect involvement in gene regulation [1, 5]. Accounting for strand information is critical, and this is inherently addressed by the bidirectional nature of DFMs, as promoter complexes are bidirectional [1, 5, 39]. We further benchmark traditional deep learning approaches, including the iPro-WAEL ensemble and the sequence-only iChrom-deep-focal model using default parameters, and observe consistently lower performance compared to the DFMs (Appendix D, Supplementary Table S3) [16, 24].

We explored an alternative approach (Appendix E: Supplementary Fig. S2) by segregating the data into CpG and non-CpG promoter groups which do not show better performance (Supplementary Table S4).

Hence, we built Models A and B for each tissue (Fig. 1: Modules I and II).

- Model A: general promoter biology
- Model B: decipher TSp code

We observe better performance with Model A (F1 = 0.85–0.95) as label 0 are random non-promoter sequences across the human genome (hg38), relative to Model B where label 0 are non-TSp sites matched for GC content and repeat patterns (F1 ∼ 0.8) for 3k length. Model A focuses on finding general tissue-wise promoter logic whereas Model B finds TSp promoter logic highly specific to the tissue of interest. We then applied these models to the xAI motif discovery module (Fig. 1B: Module III, Fig. 2B).

### Deciphering brain regulatory language and its’ disease relevance

We analyzed models A and B separately, and the logic here is to find the B ∩ A^c^ tokens/motifs, so we get the unique motifs regulating tissue specificity. We follow the attention analysis as described in Methods “Attention-based motif discovery.

**Figure 3.**
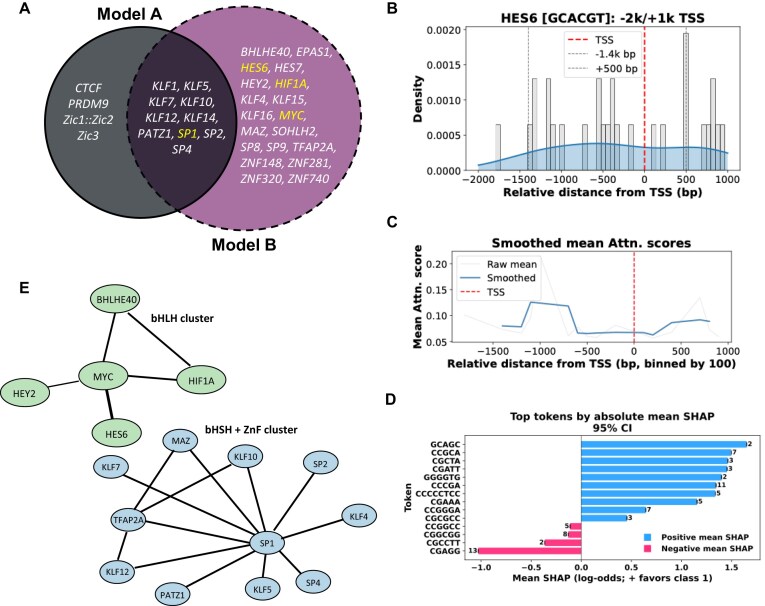
(**A**) Overlap of top significant motifs in brain identified across Models A and B found among JASPAR hits. (**B**) Density of GES6 transcription-factor motif occurrences relative to the TSS (red), with upstream −1.4 kb and downstream +500 bp regions indicated. (**C**) Smoothed mean attention scores relative to the TSS, highlighting enrichment regions. (**D**) Top sequence tokens ranked by absolute mean SHAP values with 95% confidence intervals. Token counts shown above each bar. (**E**) Two biclusters of brain-specific Model B and the TF–TF interactors. The basic helix–loop–helix (bHLH) cluster corresponds to motif “GCACGT” and basic helix–span–helix (bHSH) + ZnF (zinc finger) cluster corresponds to motif “GGCCTGGGGGCGGGG”.

In Model B, we identified 82 unique significant motifs for motif significance testing, of which 69 matched targets in the JASPAR database, including 29 from vertebrates. To ensure robustness, we extracted attention scores only from correct model predictions. Among the motifs identified, 20 corresponded to C2H2 zinc finger factors (ZNFs), 8 to basic helix–loop–helix (bHLH) factors, and 1 to a basic helix–span–helix (bHSH) factor. We also observed the “*GGGGCGGGG*” repeat to be significantly enriched in both Models A and B, prompting further investigation of its biological/ disease relevance. Motifs mapping to known TFs were identified, as illustrated (Fig. 3A).

Models A and B share 10 TF motifs from JASPAR, with *SP1* emerging as one of the experimentally validated clinical hits. It is listed in the TF Marker [20] as relevant to cancer cells in glial populations (Appendix F3: Supplementary Tables S5 and S6). Appendix F2: Supplementary Fig. S4 highlights that Model B concentrates on SP1 motifs, with elevated attention and SHAP signals across the −1600 to +500 bp region, indicating its role in TSS-proximal specificity. In contrast, Model A distributes *SP1* motifs more evenly, emphasizing core and proximal promoters and reflecting broader brain promoter features (Appendix F1: Supplementary Fig. S3).

*SP1*, a ubiquitous TF, binds to gene promoters and plays a central role in transcription. This TF has also been found to be cloned into the FLEX calling cards system for cell type–specific applications and is known to interact with gene *BRD4*. Analysis from TransTExdb shows that *SP1* expression is predominantly widespread (like housekeeping) or lowly expressed transcripts. It was striking that *SP1* is a significant marker of gliomas in the brain. Also, *BRD4*-mediated *MYC* TF degradation has significant translational implications [40]. Additionally, the *Sp1–TIMP1* axis has been reported as a potent biomarker for evaluating immune cell infiltration at tumor sites and for tracking malignant progression in glioblastoma of the brain [41]. This highlights the critical role of these TFs in normal and disease biology.

Another significant motif “*GCACGT*” enriched among Model B ∩ A^c^ motifs, maps to 2 TFs of interest, *HES6* (Fig. 3B–D) and *MYC*, both belonging to the bHLH class of TFs. Most of *HES6*’s transcripts from TransTEx are non-TSp except *ENST00000409356* which is specific to the pituitary gland. Figure 3B shows the distribution of attention scores, which is enriched across the 3k bp window around −1400 to 500 bp. Smoothed attention profiles show elevated attention scores in the same region, a few bp away from the TSS, suggesting the value of this model’s predictions in looking at longer regions around TSS (Fig. 3C). SHAP analysis further focuses on similar repeats like *CGA, GC*-rich tokens, particularly *HES6*-like TFs, as key drivers of positive classification. This supports the biological relevance of the learned features and demonstrated that the model captures distributed, motif-level regulatory signals (Appendix F2). Our findings demonstrate that SHAP analysis, high performance of transformer-based architectures and biological interpretability. By assigning a “fair” contribution score to each k-mer based on its marginal impact across all possible genomic contexts, we have moved beyond the “black-box” limitations often associated with deep learning in genomics. The concentration of high SHAP values around known TFBS suggests that TSProm is not merely overfitting to sequence noise but is instead identifying valid biological signatures. The variation in motif importance between tissues such as, the unique reliance on specific GC-rich clusters in the Liver underscores the model’s ability to learn tissue-specific “regulatory languages.” These results provide a robust foundation for future studies into how non-coding variants might disrupt these high-impact motifs, potentially leading to TSp disease phenotypes.

To further decipher the *cis-*regulatory modules underlying brain specificity, we applied spectral biclustering to the significant motifs identified by TOMTOM, revealing two major clusters: a bHLH cluster centered on the *GCACGT* motif (HIF/MYC/HES factors) involved in neural differentiation and a bHSH* + * ZnF cluster with GC-rich motifs bound by KLF/SP/ZnF transcription factors that regulate promoter architecture and neural gene expression [19, 42–44]. Figure 3E shows the TF–TF interactions from RegNetwork Database 2025 [45].

Direct validation using ENCODE ChIP-seq experiments [22] shows that TSProm attention-enriched regions are significantly enriched over the 3 kb background in brain. While traditional motif analysis identifies many generic motifs (131; only 7 overlapping), TSProm yields a focused set (19 TFs) enriched for brain-relevant and clinically implicated regulators (e.g. *MYC, HIF1A*, and *HES6*). Further, we observe that the seven common TFs do not show significant known clinical relevance as reported by KnockTF or TFMarker as compared to *MYC, HIF1A*, and *HES6*, which have clinical relevance as reported validating the application of TSProm’s xAI-based interpretability framework to understand brain specificity in the context of gene regulation and disease. Another reason ENCODE peaks identify a larger number of associated TFs is that these datasets are typically tissue-enriched rather than TSp, leading to the inclusion of broadly active TFs not uniquely relevant to brain regulation.

To conduct peak-wise quantitative validation, we analyze the significant attention regions predicted by TSProm models for brain against a background of the entire 3k length using ENCODE ChIP-seq experiments. Traditional motif analysis on 3 kb brain sequences identified 131 significant JASPAR motifs but showed minimal overlap (7 TFs) with TSProm predictions. In contrast, TSProm yielded a smaller, more selective set (19 TFs) enriched for brain-relevant and clinically implicated regulators (e.g. *MYC, HIF1A*, and *HES6*). This highlights the advantage of the attention-based TSProm framework in prioritizing tissue-specific regulatory motifs over generic TF signals. We also find that among the top significant peaks we find important TFs belonging to both *cis-*regulatory modules of brain like bHLH and bHSH + ZnF clusters (Appendix F4: Supplementary Fig. S5).

### Testis specific regulatory language

To validate the framework for another tissue, we focused on the testis due to the availability of data and its biological importance. We analyzed transcription factors enriched in testis-specific promoters and identified several factors that were uniquely overrepresented in this tissue, including *RFX1, RFX2, RFX3, Patz1, SP2, SP5*, and multiple members of the ZnF family.

Among the identified factors, *RFX2* stands out as a key regulatory gene involved in spermiogenesis—the process by which spermatids mature into sperm [46]. *RFX2*-binding sites were also found to be enriched in super-enhancer regions associated with glioblastoma, suggesting that this factor plays a broader role in coordinating complex transcriptional programs beyond the testis [47]. These distal enhancer elements were effectively captured by the TSProm framework, demonstrating its ability to identify both proximal and distal sequence features that drive tissue-specific regulation (Appendix G: Supplementary Fig. S6).

Biclustering of testis-specific promoter motifs revealed nine distinct regulatory clusters, with the best-performing with a silhouette score 0.803of spectral biclustering. The analysis highlights a ZnF-dominated regulatory landscape, often coupled with Forkhead, MADS-box, and bHLH motifs. Together, these modules suggest combinatorial control by *CTCF, SP/KLF*, and *PRDM9* families shaping testis-specific promoter activity (Appendix G: Supplementary Table S7) [19].

### Language of the global tissue specificity

**Figure 4.**
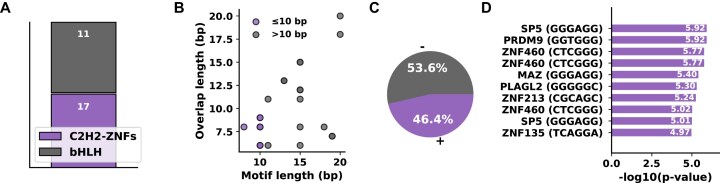
Landscape of overall tissue specificity. (**A**) Distribution of motif hits across transcription factor classes in vertebrates. (**B**) Comparison of motif overlaps grouped as short (≤10 bp, purple) and long (>10 bp, gray). (**C**) Strandedness of motif matches. (**D**) Top enriched human motifs ranked by significance; *y*-axis indicates transcription factors (consensus sequences).

Among the significant JASPAR hits for TF motifs, we find most are involved in C2H2–ZnF TF domain and bHLH (Fig. 4A and Appendix H) C2H2–ZnFs are the largest family of TFs and are relevant throughout development, gene regulation, and disease biology. Fedotova *et al*. [48] quote that, C2H2 ZNFs, a family of proteins are the largest but poorly explored family among eukaryotic TFs. Genes encoding this TF contain proteins that make up ∼40% of all human TF genes [49]. In dysregulation, these proteins regulate transcription of downstream genes involved in proliferation, apoptosis, and cell invasion thereby causing cancer invasion [50]. According to KnockTF 2.0, we find *ZNF213, ZNF385* reported in adenocarcinoma and leukemia cell lines [21].

Similarly, we identify *MYF5* as a relevant TF Protein marker from TF-Marker, motif “*ACAGCTGT*” important in embryonic stem cells that can remodel chromatin in developing embryonic mesenchymal cells for myogenesis [20] (Appendix H: Supplementary Table S8). When stratifying motifs by length, we found that longer motifs (>10 bp) showed greater overlaps with target motifs than shorter motifs (≤10 bp) (Mann–Whitney *U* = 42.0, *P* = 0.012) (Fig. 4B). Also, there are more negative (−) stranded TF motifs and the top human TSp TFs are summarized Fig. 4C). We observe *ZNF460* and *SP5* repeats in the chart, suggesting multiple significant motif patterns (Fig. 4D).

TF *ZNF460* is significantly upregulated in gastric, colorectal cancers and promotes metastasis visa the *JAK/STAT3* pathway [51, 52]. *SP5*, a *C2H2 ZnF* TF, modulates *WNT* signaling by balancing transcriptional activation and repression through distinct functional domains. Its elevated expression in hepatocellular, gastric, and colon cancers highlights its role as a context-dependent regulator of tumorigenesis [53, 54].

Overall, TSProm attention mechanism identifies significant and major TFs contributing to understanding the sequence semantics of major TFs in humans and can be extended to other species.

## Discussion

The TSProm framework represents a significant advancement in applying DFMs to understand the regulatory mechanisms modeling tissue specificity, which can then be implemented toward disease insights. By leveraging transformer architectures to analyze long-range promoter sequences up to 3 kb upstream and 1 kb downstream of TSS, our framework identifies clinically relevant TFs that traditional proximal and/or core promoter analyses often do not emphasize on. The framework’s ability to distinguish between general promoter biology: *Model A* and TSp regulatory mechanisms: *Model B* provides a systematic approach to dissect the molecular basis of healthy tissues, study its relevance in disease biology, particularly in complex diseases like cancer and neurodegeneration, where there is known TSp dysregulation of normal regulatory programs [2, 55, 56].

Our analysis of brain-specific regulatory elements reveals key TFs with direct clinical implications for neurological diseases. We identify two dominant clusters of brain-specific regulatory modules which code for a neural differentiation cluster and a promoter architecture and neuronal expression regulator cluster. The identification of *SP1* as a central regulator of brain tissue specificity is particularly significant given its established role as a biomarker for glioma and progression of neurodegeneration. The motifs of significance are also validated with xAI module and SHAP analysis which show CGA and GC-enriched motifs as positive contributors to the TSp sequences. The *SP1–TIMP1* axis identified through our attention-based motif discovery has been experimentally validated as a predictor of immune cell infiltration and malignant progression in glioblastoma, demonstrating the translational potential of our approach. Similarly, the enrichment of *HES*6 and *MYC* motifs in brain-specific promoters provides mechanistic insights into neuroblastoma and glioblastoma pathogenesis as these factors are known to be dysregulated in aggressive brain cancers. This validates TSProm’s capacity to identify regulatory elements that transition from normal tissue specification to disease-driving mechanisms. We also test the framework and validate on testis TSp regulatory landscape and identify a key regulatory of spermiogenesis which also had relevance in an aggressive brain cancer like glioblastoma [46, 47].

We find predominance of *C2H2* ZnF proteins TFs in global TSp landscape through our attention-based module. These TFs comprise ∼40% of the human transcriptome yet remain poorly characterized, representing possible therapeutic targets. Our identification of *ZNF460* and *SP5* as significantly enriched TF motifs, with their roles in gastric, colorectal, and hepatocellular cancers, suggests that TSp regulatory networks may be broadly dysregulated in multiple cancer types. Cross-referencing these TFs with experimentally validated databases like TF-Marker and KnockTF 2.0 provides validation for the fine-tuned models’ interpretability module. The framework’s xAI components, including attention-based motif discovery and model-agnostic SHAP analysis, ensure validations of predictions.

While TSProm provides a robust and interpretable framework for modeling TSp promoter regulation, it is currently focused on promoter sequence contexts based on the TSS and bulk tissue level annotations. This may not fully capture the cell type specific effects due to the heterogeneity of tissues considered in this analysis. In addition, the TF and promoter *cis*-regulatory modules inferred from attention-based and SHAP analyses are associative and will benefit from future targeted experimental validations to establish direct regulatory causality. Their functional relevance can be tested using reporter gene assays to assess regulatory activity, ChIP-seq to evaluate TF binding, targeted mutagenesis followed by expression analysis, and ATAC-seq to identify open chromatin regions. Finally, the applicability of the framework is influenced by the availability and quality of transcriptomic annotations across tissues and can be extended to more primate species. This will enable broader generalization to additional biological contexts as more high-resolution datasets become available.

As DFMs continue to evolve, TSProm provides a robust pipeline for translating genomic sequences around promoters into actionable insights for precision medicine, with the potential to transform our outlook, to tackle complex genetic diseases through the lens of tissue specificity and its disruption in disease states. We further anticipate the application of this framework to other tissues and cell types contingent on data availability.

## Supplementary Material

lqag050_Supplemental_File

## Data Availability

TSProm code and Model weights: https://doi.org/10.6084/m9.figshare.30747257 Isoform expression data: GTEx (see GTEx Consortium 2015, 2020). Human TransTEx groupings and code: Described in Surana *et al*. (2024) and available at TransTExdb. Mouse BodyMap dataset: Available from NCBI BioProject PRJNA375882 (Li *et al*., 2017). Hugging Face DNA language models (Fishman *et al*., 2025; Zhou *et al*., 2021; Dalla Torre *et al*., 2025): AIRI-Institute/gena-lm-bert-large-t2t AIRI-Institute/gena-lm-bigbird-base-t2t InstaDeepAI/nucleotide-transformer-500m-1000g jaandoui/DNABERT2-AttentionExtracted zhihan1996/DNABERT-2–117M
