# Supplementary material for: TSProm: deep learning framework to predict tissue-specific regulatory logic
Source: NAR Genom Bioinform. 2026 Jun 3;8(2):lqag050. doi: 10.1093/nargab/lqag050 (PMC13233143; doi:10.1093/nargab/lqag050)
Supplement: lqag050_Supplemental_File [file lqag050_supplemental_file.docx]

**TSProm: Deep learning framework to predict Tissue-specific regulatory logic**

Pallavi Surana^1^, Pratik Dutta^1^, Nimisha Papineni^1^, Rekha Sathian^1^, Zhihan Zhou^2^,

Han Liu^2^ and Ramana Davuluri^1,*^

**Supplementary Information**

**Appendix A: Tissue choice for *TSProm***

We selected testis, brain, and liver because these tissues contained the highest number of transcripts, from which we extracted regions around the transcription start site (TSS). To address reviewer concerns about the applicability of *TSProm* to other tissues, we extended the analysis to two additional tissue transcript sets: muscle and spleen. Ovary was not included since the number of transcripts in the human data was less than~100, which was insufficient for robust training. We aim to have at least ~1,000 transcripts in the positive set to reduce the risk of overfitting for these large language models.

We used *MMseqs2* to remove sequences with more than ~80% similarity, which reduced the number of unique transcripts available for each tissue. The final datasets were divided into training, validation, and test subsets to ensure balanced evaluation.

**Supplementary Table 1:** Transcript counts by tissue and source in mouse and human TransTEx datasets

| **Tissue** | **Mouse TransTEx** | **Human TransTEx** | **Human + Mouse** |
| --- | --- | --- | --- |
| testis | 9045 | 12459 | 21504 |
| brain | 2493 | 612 | 3105 |
| liver | 427 | 774 | 1201 |
| ovary | 1032 | 71 | 1103 |
| muscle | 264 | 338 | 602 |
| spleen | 348 | 187 | 535 |
| lung | 242 | 142 | 384 |
| bone marrow, blood, blood vessel | 156 | 204 | 360 |
| heart | 219 | 126 | 345 |
| adrenal gland | 150 | 117 | 267 |
| small intestine | 143 | 57 | 200 |
| vesicular/ salivary gland | 10 | 76 | 86 |
| stomach | 50 | 2 | 52 |
| Total (13 common tissues) | 14579 | 15165 |  |

*Totals mapped to Ensembl gene names: Mouse TransTEx: 17,320; Human TransTEx: 17,619.*

**Appendix B: Rationale for sequence length and TSS-centered regions**

Transcription factor binding sites (TFBS) located near the TSS typically exert the strongest influence on transcriptional activity. These sites can act as activators or repressors depending on the transcription factor and its interaction with other cofactors. Although more distal TFBS also play roles in gene regulation, their effects are generally mediated through cooperative interactions or chromatin looping.


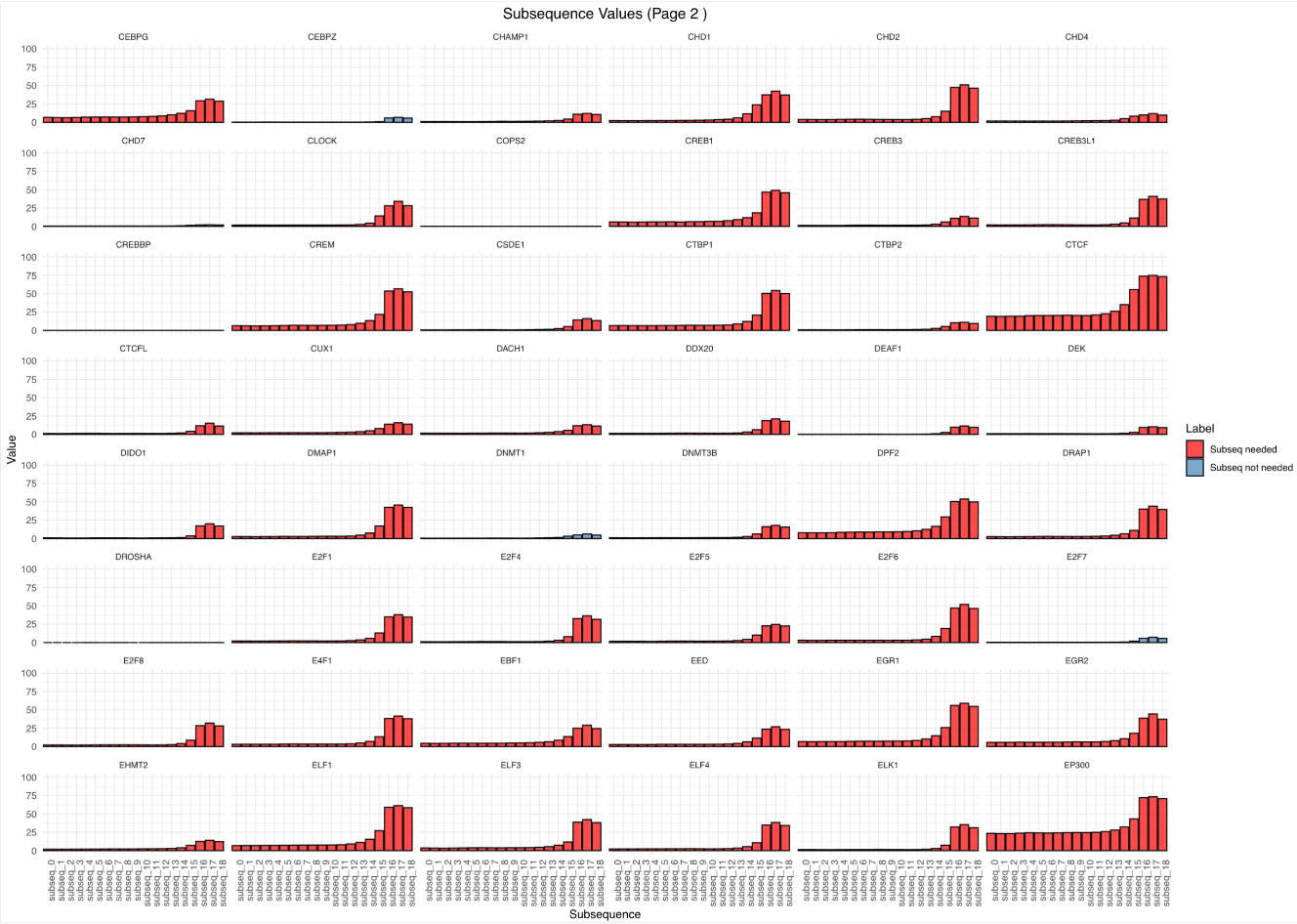


**Supplementary Figure 1:** Illustrative example showing the enrichment of TFBS near the TSS. The highest density occurs within ~3 kb upstream and 1 kb downstream of the TSS, highlighting the biologically relevant region for promoter modeling.

To determine the most biologically meaningful sequence length around the TSS, we analyzed transcription factor binding data and histone mark profiles from the ENCODE consortium. The enrichment of TFBS around the TSS indicates that most experimentally validated sites occur within approximately 3 kb upstream and 1 kb downstream of the TSS. This range represents a core promoter-proximal region where transcriptional regulation is most direct, while still encompassing some distal elements that contribute to gene-specific expression [1-3].

Based on this biological evidence, we focused on modeling promoter sequences in windows extending several kilobases upstream and downstream of the TSS. This design allows us to capture both proximal promoter elements and longer-range regulatory signals.

**Appendix C: DNABERT2 model parameters and the evaluation metrics used**

The model is based on a bidirectional Transformer encoder architecture similar to BERT, with 12 encoder layers, 12 attention heads, and a hidden dimension of 768, totaling approximately 117 million trainable parameters. Contextual embeddings are produced for each token, while the embedding corresponding to the special [CLS] token is used as a global sequence representation for downstream prediction tasks.

To enable robust handling of long genomic sequences, DNABERT-2 replaces standard learned positional embeddings with Attention with Linear Biases (ALiBi). This mechanism introduces distance-dependent biases directly into the attention scores, progressively discouraging interactions between distant tokens. By decoupling positional information from fixed embedding tables, ALiBi allows the model to extrapolate to sequences longer than those observed during pretraining, while preserving the standard quadratic attention formulation.

For efficient fine-tuning and inference on long sequences, DNABERT-2 supports FlashAttention, which optimizes memory access patterns to reduce both runtime and memory consumption without altering attention outputs. In addition, Low-Rank Adaptation (LoRA) is employed as a parameter-efficient fine-tuning strategy, introducing a small number of additional trainable parameters into attention layers. This approach significantly lowers computational and memory requirements while maintaining performance comparable to full model fine-tuning.

There are the summary of model metrics used to evaluate the fine-tuning of the DFMs used.

- $\text{Accuracy}=\frac{TP+TN}{TP+TN+FP+FN}$
- $\text{Precision}=\frac{TP}{TP+FP}$
- $\text{Recall}=\frac{TP}{TP+FN}$
- $F1=\frac{2\times\text{Precision}\times\text{Recall}}{\text{Precision}+\text{Recall}}$
- $MCC=\frac{TP\times TN-FP\times FN}{\sqrt{(TP+FP)(TP+FN)(TN+FP)(TN+FN)}}$

**Appendix D: Benchmarking with GENA-LM, DNABERT2 and Nucleotide Transformer**

The table below extends Figure 2 by reporting per–tissue results across sequence lengths (2k, 3k and 4k bp) and learning rate settings (see the *Run* column). The best performance was obtained when using Wide or Null as the negative class. *Wide* (widespread) promoters typically correspond to housekeeping or basal regulatory functions, whereas *Null* denotes minimal expression in adult normal tissues. These observations motivated our choice of *Wide/Null* as the negative set for fine-tuning, to better capture the regulatory logic of TSp promoter regions.

Unless otherwise specified, models were trained with weight decay = 1 x 10-2, warmup steps=10, epochs=10, and gradient accumulation steps =1. We used sequence length in {2k, 3k, 4k} bp and learning rates in a small grid covering [5 × 10⁻⁶, 7 × 10⁻⁶] and [2 × 10⁻⁵, 3 × 10⁻⁵]; the exact setting for each run is indicated in the table.

**Supplementary Table 2:** Performance comparison DNABERT2, GENA-LM and NT (500m--1000G) models across tissues and sequence lengths. Metrics include Precision, Recall, F1-score, MCC, and Accuracy.

| **Length (bp)** | **Tissue** | **TSp site** | **Run LR** | **Precision** | **Recall** | **F1** | **MCC** | **Accuracy** |
| --- | --- | --- | --- | --- | --- | --- | --- | --- |
| **DNABERT2** | | | | | | | | |
| 2k | Brain | null | lr5e-6 | 80.26 | 80.23 | 80.22 | 60.49 | 80.23 |
| 3k | Brain | null | lr3e-6 | 81.21 | 81.19 | 81.19 | 62.4 | 81.19 |
| 4k | Brain | null | lr3e-6 | 80.07 | 80.06 | 80.06 | 60.13 | 80.06 |
| 2k | Liver | wide | lr5e-6 | 79.57 | 78.1 | 77.82 | 57.65 | 78.1 |
| 3k | Liver | wide | lr5e-6 | 84.77 | 84.71 | 84.7 | 69.48 | 84.71 |
| 4k | Liver | wide | lr7e-6 | 80.83 | 80.58 | 80.54 | 61.41 | 80.58 |
| 2k | Testis | wide | lr7e-6 | 76.73 | 76.27 | 76.16 | 52.99 | 76.27 |
| 3k | Testis | wide | lr7e-6 | 76.97 | 75.83 | 75.56 | 52.79 | 75.83 |
| 4k | Testis | wide | lr3e-5 | 77.37 | 77.2 | 77.16 | 54.57 | 77.2 |
| 2k | Muscle | null | lr3e-6 | 67.97 | 67.31 | 67 | 35.27 | 67.31 |
| 3k | Muscle | null | lr7e-6 | 67.97 | 67.31 | 67 | 35.27 | 67.31 |
| 4k | Muscle | null | lr5e-6 | 71.15 | 71.15 | 71.15 | 42.31 | 71.15 |
| 2k | Spleen | null | lr7e-6 | 74.48 | 73.96 | 73.82 | 48.43 | 73.96 |
| 3k | Spleen | low | lr7e-6 | 75.05 | 72.92 | 72.33 | 47.92 | 72.92 |
| 4k | Spleen | null | lr5e-6 | 78.31 | 77.08 | 76.83 | 55.38 | 77.08 |
| **GENA-LM** | | | | | | | | |
| 2k | Brain | null | lr2e-5 | 76.26 | 82.64 | 79.32 | 57.11 | 78.46 |
| 3k | Brain | null | lr2e-5 | 79.08 | 82.64 | 80.82 | 60.83 | 80.39 |
| 4k | Brain | null | lr5e-6 | 78.41 | 88.75 | 83.26 | 64.87 | 82.15 |
| 2k | Liver | wide | lr5e-6 | 78.2 | 85.95 | 81.89 | 62.29 | 80.99 |
| 3k | Liver | tenh | lr5e-6 | 72.78 | 95.04 | 82.44 | 62.5 | 79.75 |
| 4k | Liver | wide | lr5e-6 | 75.4 | 78.51 | 76.92 | 52.94 | 76.45 |
| 2k | Testis | tenh | lr2e-5 | 75.86 | 74.52 | 75.19 | 50.82 | 75.41 |
| 3k | Testis | wide | lr5e-6 | 77.7 | 75.83 | 76.75 | 54.08 | 77.03 |
| 4k | Testis | tenh | lr2e-5 | 72.47 | 79.68 | 75.91 | 49.67 | 74.71 |
| 2k | Muscle | low | lr5e-6 | 54.02 | 90.38 | 67.63 | 18.2 | 56.73 |
| 3k | Muscle | low | lr3e-4 | 50 | 100 | 66.67 | 0 | 50 |
| 4k | Muscle | low | lr2e-5 | 52.5 | 40.38 | 45.65 | 3.95 | 51.92 |
| 2k | Spleen | low | lr2e-5 | 75.61 | 64.58 | 69.66 | 44.22 | 71.88 |
| 3k | Spleen | low | lr5e-6 | 80 | 66.67 | 72.73 | 50.71 | 75 |
| 4k | Spleen | low | lr2e-5 | 57.63 | 70.83 | 63.55 | 19.26 | 59.38 |
| **NT (500m–1000G)** | | | | | | | | |
| 2k | Brain | low | lr5e-6 | 68.14 | 66.98 | 66.44 | 35.1 | 66.98 |
| 3k | Brain | null | lr5e-6 | 71.36 | 71.21 | 71.16 | 42.57 | 71.21 |
| 4k | Brain | null | lr5e-6 | 74.39 | 74.04 | 73.95 | 48.42 | 74.04 |
| 2k | Liver | low | lr2e-5 | 62.25 | 62.22 | 62.2 | 24.47 | 62.22 |
| 3k | Liver | null | lr2e-5 | 59.63 | 59.55 | 59.47 | 19.18 | 59.55 |
| 4k | Liver | null | lr2e-5 | 62.6 | 62.5 | 62.42 | 25.1 | 62.5 |
| 2k | Muscle | low | lr3e-4 | 52.44 | 51.92 | 49.22 | 4.34 | 51.92 |
| 3k | Muscle | low | lr3e-4 | 50 | 50 | 49.33 | 0 | 50 |
| 4k | Muscle | low | lr2e-5 | 53.99 | 53.85 | 53.42 | 7.84 | 53.85 |
| 2k | Spleen | low | lr2e-5 | 60.43 | 60.42 | 60.4 | 20.85 | 60.42 |
| 3k | Spleen | low | lr2e-5 | 65.8 | 65.62 | 65.53 | 31.42 | 65.62 |
| 4k | Spleen | low | lr2e-5 | 55.21 | 55.21 | 55.2 | 10.42 | 55.21 |
| 2k | Testis | low | lr5e-6 | 53.44 | 53.25 | 52.6 | 6.69 | 53.25 |
| 3k | Testis | low | lr5e-6 | 57.04 | 56.25 | 54.99 | 13.27 | 56.25 |
| 4k | Testis | low | lr5e-6 | 59.15 | 59 | 58.83 | 18.15 | 59 |

The table below extends Figure 2 by reporting per–tissue results across sequence lengths (2k, 3k and 4k bp) and the models default parameters were used. The best performance was obtained when using Wide or Null as the negative class. *Wide* (widespread) promoters typically correspond to housekeeping or basal regulatory functions, whereas *Null* denotes minimal expression in adult normal tissues. The goal is to compare against traditional deep learning methods to evaluate the performance of the TSp datasets to validate the need for foundation models.

**Supplementary Table 3:** Performance comparison Ensemble method iPro-WAEL [4] and IChrom-Deep-focal [5] models across tissues and sequence lengths. Metrics include Area Under Curve, Accuracy, and MCC. For IChrom-Deep-focal we ran the sequence only version of the model because we had no chromatin information available in this tissue specific dataset we curated.

| **Length (bp)** | **Tissue** | **Condition** | **AUC** | **Accuracy** | **MCC** |
| --- | --- | --- | --- | --- | --- |
| **iPro-WAEL** | | | | | |
| 2000 | brain | null | 79.94 | 72.69 | 45.4 |
| 3000 | brain | null | 80.09 | 71.44 | 42.94 |
| 4000 | brain | null | 79.8 | 72.69 | 45.48 |
| 2000 | liver | wide | 80.75 | 72.7 | 45.65 |
| 3000 | liver | wide | 81.24 | 73.03 | 46.07 |
| 4000 | liver | wide | 78.51 | 73.09 | 46.2 |
| 2000 | testis | wide | 71.34 | 65.88 | 31.91 |
| 3000 | testis | wide | 73.03 | 67.31 | 34.77 |
| 4000 | testis | wide | 73.16 | 68.02 | 36.07 |
| **IChrom-Deep-focal** | | | | | |
| **Length (bp)** | **Tissue** | **Condition** | **AUC** | **F1** | **MCC** |
| 2000 | Brain | null | 65.46 | 66.67 | 20.56 |
| 3000 | Brain | null | 67.19 | 66.67 | 29.34 |
| 4000 | Brain | null | 62.58 | 66.67 | 12.03 |
| 2000 | Liver | wide | 74.46 | 66.96 | 41.08 |
| 3000 | Liver | wide | 72.21 | 66.67 | 19.57 |
| 4000 | Liver | wide | 70.34 | 66.67 | 6.77 |
| 2000 | Testis | wide | 69.82 | 66.67 | 25.56 |
| 3000 | Testis | wide | 69 | 66.67 | 25.5 |
| 4000 | Testis | wide | 69.16 | 66.67 | 24.98 |

**Appendix E: Other pre-training and fine-tuning approaches which did not improve model performance**

*E.1 Motivation*


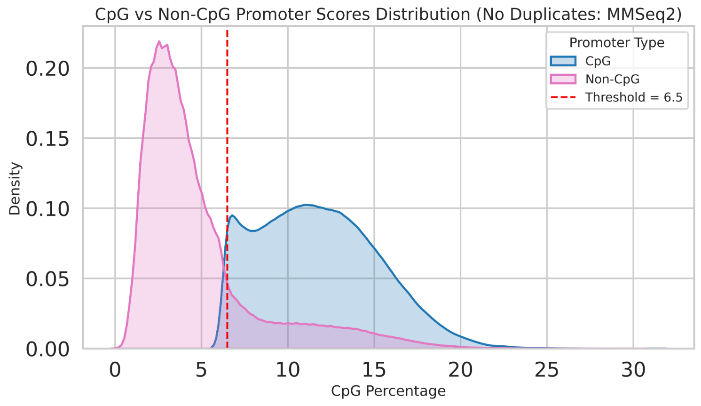


**Supplementary Figure 2:** 201bp windows along 6k length around TSS (5k, 1k, TSS) CpG score distribution across the dataset used for pretraining.

CpG and non-CpG promoter profiles differ in their sequence and regulatory properties, motivating us to test whether separating these groups could improve model performance [6, 7]. We therefore hypothesized that pretraining two DNABERT2 models - one on CpG promoters and another on non-CpG promoters - followed by fine-tuning might better capture their distinct features. However, this split introduced strong tissue imbalances; for example, in liver the CpG subset is small (~250 promoters) compared with non-CpG. Consequently, the effective sample sizes were much smaller than in our original setup, which likely explains the poor results we observed. It is also known that, in general, TSp regions contain more non-CpG promoters than other expression groups. These findings are consistent with prior reports linking non-CpG promoters to TSp genes and CpG-rich promoters to housekeeping genes that are constitutively active across tissues [6, 8].

*E. 2 Pretraining and Fine-tuning:*

We pre-trained the models using 6 kb sequences centered around TSS from 30 species, including primates (for example, C. syrichta, A. nancymaae, G. gorilla, H. sapiens, M. mulatta, M. nemestrina, N. leucogenys, O. garnettii, P. abelii, P. anubis, P. paniscus, P. troglodytes, R. bieti, R. roxellana, R. norvegicus, S. boliviensis) and rodents (for example, H. griseus, C. lanigera, I. tridecemlineatus, J. jaculus, M. murinus, M. caroli, M. musculus, M. pahari, M. spicilegus, M. spretus, N. galili, P. bairdii, R. norvegicus, M. fascicularis), obtained via the biomaRt package (version 2.60.1) [9]. However, during fine-tuning, the models did not show substantial improvement, as summarized in table below. For fine-tuning, we further divided promoters into CpG-rich and non-CpG groups using a 6.5% CpG-content cutoff, computed over sliding 201 bp windows across the TSS-centered regions, but this stratification did not result in significant performance gains.

**Supplementary Table 4:** Results after further pretraining on CpG vs. non-CpG promoters, followed by fine-tuning

| **Condition** | **Size** | **Run LR** | **Precision** | **Recall** | **F1** | **MCC** | **Accuracy** |
| --- | --- | --- | --- | --- | --- | --- | --- |
| **Non-CpG Further Pretrained Model** | | | | | | | |
| brain_null | 2000 | lr5e-6 | 82.95 | 82.89 | 82.83 | 65.84 | 82.83 |
| liver_wide | 3000 | lr3e-6 | 82.12 | 82.05 | 81.94 | 64.17 | 81.94 |
| liver_wide | 3000 | lr5e-6 | 81.61 | 81.57 | 81.48 | 63.18 | 81.48 |
| liver_wide | 3000 | lr7e-6 | 81.47 | 81.49 | 81.48 | 62.96 | 81.48 |
| brain_null | 2000 | lr7e-6 | 81.14 | 81.08 | 81.02 | 62.22 | 81.02 |
| **CpG Further Pretrained Model** | | | | | | | |
| brain_null | 4000 | lr5e-6 | 79.1 | 78.6 | 78.77 | 57.7 | 79.15 |
| brain_null | 4000 | lr7e-6 | 77.65 | 77.17 | 77.33 | 54.82 | 77.74 |
| brain_wide | 4000 | lr7e-6 | 76.56 | 76.5 | 76.52 | 53.05 | 76.68 |

**Appendix F: Brain specific models and clinical inference**

*F.1 Model A for Brain*

This shows that Model A shows a more evenly distribution of the identified motifs around TSS with peaks around the core and proximal promoter regions for TF *Zic3* that is uniquely present in this model and not Model B. This shows our model is able to capture general promoters as well as TSp promoter enriched tokens which imply critical role in biology.


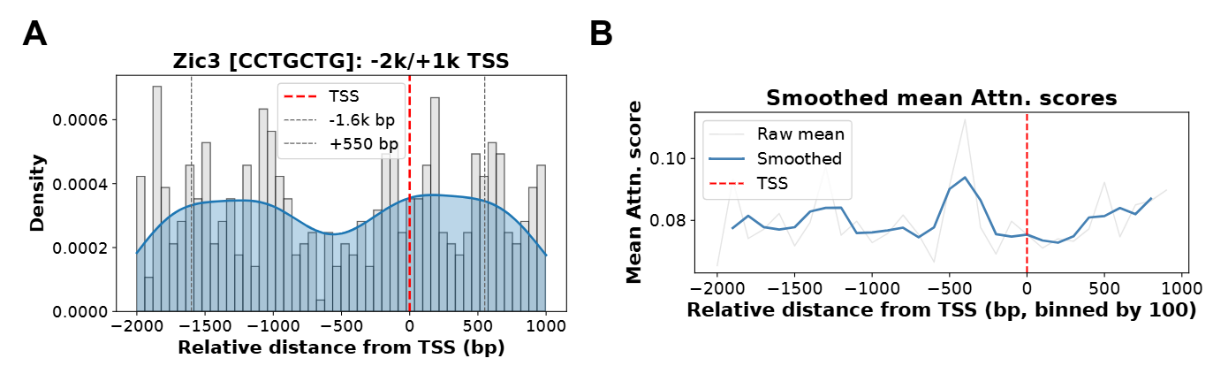


**Supplementary Figure 3:** Detailed analysis of the Zic3 transcription factor motif. (A) Density of motif occurrences relative to the TSS, with the TSS (red dashed line), upstream -1.6 kb, and downstream +550 bp indicated. (B) Smoothed mean attention scores relative to the TSS, highlighting enrichment near promoter regions.

*F. 2 Model B for Brain*

This shows that Model B shows a more evenly distribution of the identified motifs around TSS with peaks around the core and proximal promoter regions for TF SP1 that is ubiquitously present TF in both Models A and B. This shows our model can capture global brain promoter logic.


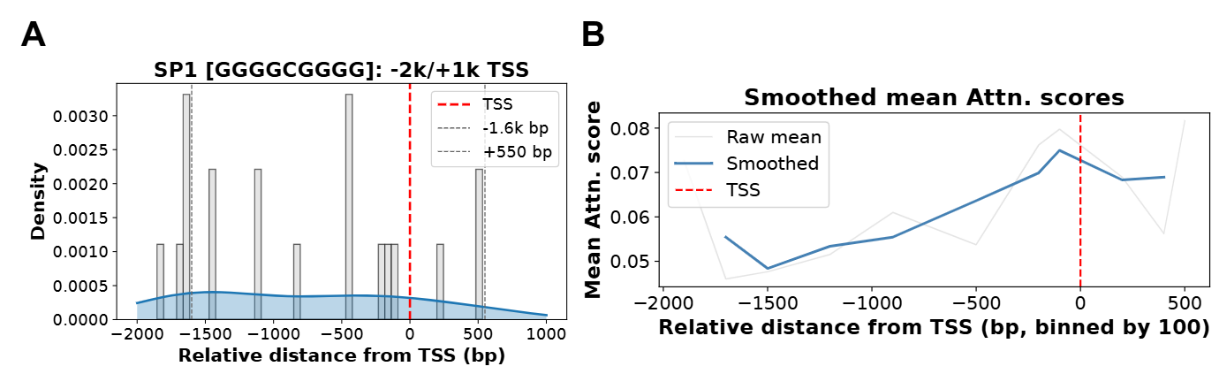


**Supplementary Figure 4:** Detailed analysis of the SP1 transcription factor motif. (A) Density of SP1 TF motif occurrences relative to TSS (red), upstream -1.6 kb, and downstream +550bp indicated. (B) Smoothed mean attention scores relative to the TSS, highlighting enrichment regions.

*F.3 Clinically relevant TFs in Model A and B*

We summarize the clinically relevant motifs for brain from KNOCK-TF 2.0 and TF-marker for brain specific TFs.

**Supplementary Table 5:** Representative hits from KnockTF 2.0 [10] showing enrichment of ‘GCACGT’ motif in glioblastoma and neuroblastoma cell lines.

| **Query ID** | **Target ID** | **Offset** | **p-value** | **E-value** | **q-value** | **Overlap** | **TF** | **Tissue** | **Cell line** |
| --- | --- | --- | --- | --- | --- | --- | --- | --- | --- |
| GCACGT | MA1493.1 | 1 | 7.46E-05 | 0.17510 | 0.0173884 | 6 | HES6 | Brain | A172  (glioblastoma) |
| GCACGT | MA1493.1 | 1 | 7.46E-05 | 0.17510 | 0.0173884 | 6 | HES6 | Brain | LN405  (glioblastoma) |
| GCACGT | MA0147.4 | 0 | 2.37E-04 | 0.55692 | 0.0291687 | 6 | MYC | Brain | BE(2)-C  (neuroblastoma) |
| GCACGT | MA0147.4 | 0 | 2.37E-04 | 0.55692 | 0.0291687 | 6 | MYC | Brain | NB1691 (neuroblastoma) |
| GCACGT | MA0147.4 | 0 | 2.37E-04 | 0.55692 | 0.0291687 | 6 | MYC | Brain | BE(2)-C  (neuroblastoma) |
| GCACGT | MA0147.4 | 0 | 2.37E-04 | 0.55692 | 0.0291687 | 6 | MYC | Brain | BE(2)-C  (neuroblastoma) |

**Supplementary Table 6:** Representative TF-Marker [11] hits showing motif mapping to SP1 and HIF1A in brain cell types.

| **Query ID** | **Target ID** | **Offset** | **p-value** | **q-value** | **Overlap** | **TF** | **Tissue** | **Cell type** | **Gene Name** |
| --- | --- | --- | --- | --- | --- | --- | --- | --- | --- |
| RGYCWVGGGGVGGGG | MA0079.5 | –6 | 3.18E-06 | 0.00293195 | 9 | SP1 | Brain | Nerve cell (normal) | SP1 |
| GCACGT | MA1106.2 | 0 | 2.63E-05 | 0.0173884 | 6 | HIF1A | Brain | Glial cell (cancer) | HIF1A |

*F.4*

*A. Comparison of the significant motifs from TSProm v/s traditional motif finding from Promoters*

To establish the significance of motif finding using traditional methods we use the sequences of 3k bp length and test the motifs for brain. We compare the similar motifs we find and summarize with Venn diagrams by running XSTREME tool [12] and reporting JASPAR2026_CORE_vertebrates_non redundant database motifs [13]. We report 131 total significant motifs from the brain-specific dataset and find only 7 common ones between TSProm Model B ∩ A’ compared to the ‘Traditional Motif Analysis’.

We report that the motifs we find by Traditional Motif Analysis are far many (total 131) compared to those reported by TSProm (19 total TFs). This can be attributed to the following reasons: I) the JASPAR database reports a broad range of TFs which are not necessarily tissue specific, II) The stringent filtering criteria and the usage of Model A and B used by the TSProm method tries to prioritize motif significance and relevance to TFs specific to brain regions only. Whereas the traditional approach might identify more generic motifs that appear across multiple contexts (**Supplementary Figure 5C)**.

Further, we observe that the 7 common TFs do not show significant known clinical relevance as reported by KnockTF or TFMarker as compared to *MYC, HIF1A* and *HES6* which have clinical relevance as reported. Hence, there is a need to use this attention-mechanism based interpretability framework to understand brain specificity better in the context of gene regulation and disease.

*B. Comparison with ENCODE ChIP-seq data*

We test the overlap of the motif regions we find among the brain, liver, and testis-specific significant motifs by checking them with ENCODE. The ENCODE dataset has ~ 4000 ChIP-seq bed files which map to ~700 experiments combined TF binding sites + histone marks. We overlap regions from ENCODE for humans among brain, testis and liver with i) all aggregated cCREs (all biosamples). Here, we compare with the significant motif regions we find from the analysis from TSProm. To validate the TSp regulatory logic, we use the regions significant in Model B, as we aim to understand both the global and local tissue regulatory logic. This approach is used because the ENCODE data is not tissue-specific but enriched in the tissue, thus helping to mitigate any biases.

For validation we consider only Transcription factor data from ENCODE and not histone marks. We scan in a 200 bp window around the motif region (+- 100bp) and report the overlaps with ENCODE ChIP peaks. We compare between background and significant TF regions. The background here is the 3k length of the transcript which is used without any thresholds. This gives us an estimate of the percentage of peaks found compared to all possible peaks within the bounds of the transcript (**Supplementary Figure 5A-B)**.


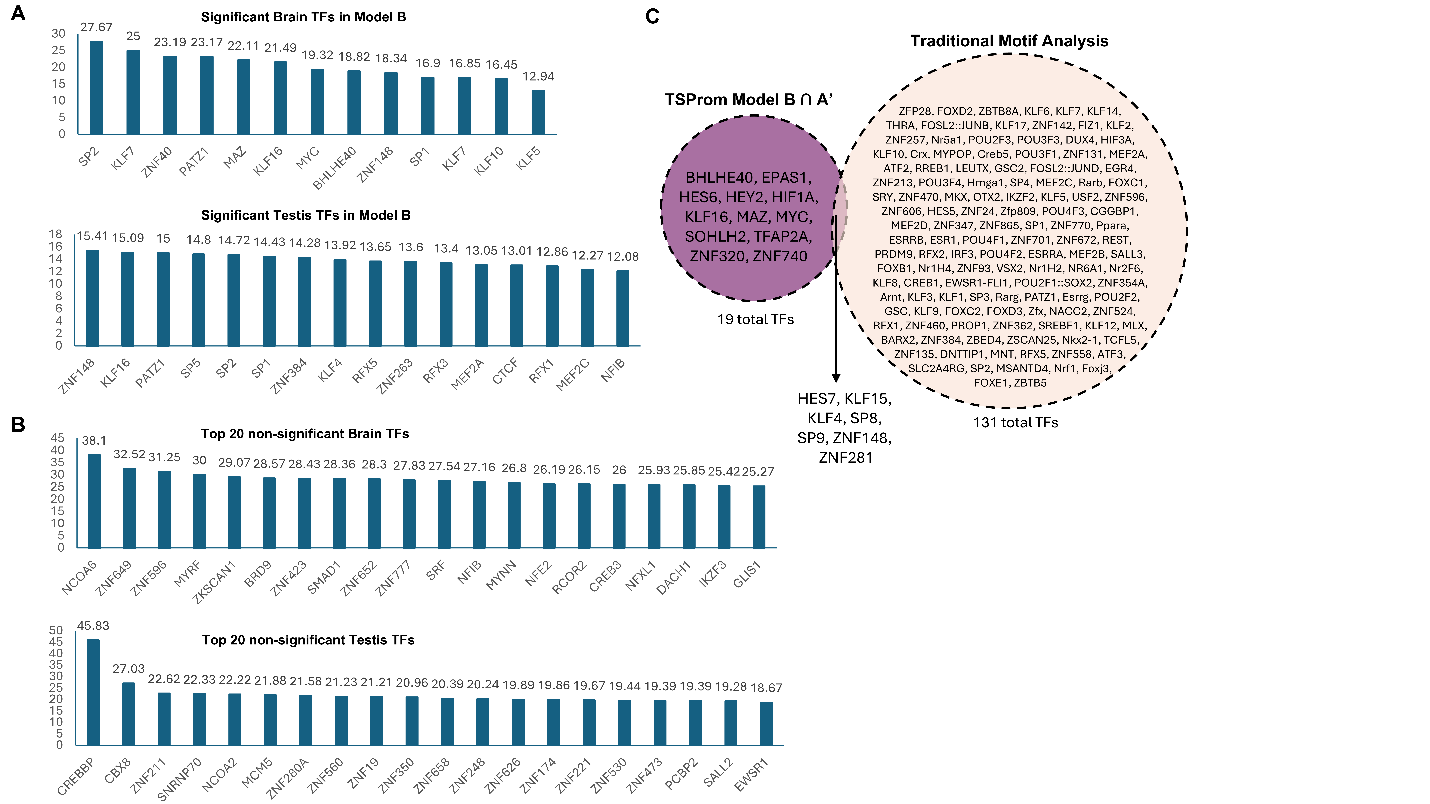


**Supplementary Figure 5:** Summary of peaks from ENCODE ChIP-seq data (A) The top significant motifs based on Model B, (B) The top 20 non-significant TFs enriched based on ENCODE ChIP-seq data are plotted. Proportions are calculated using a background which is the entire 3k bp region around the TSS without filtering for any significant regions. (C) Comparison of common TFs (7 TFs) and the total motifs in each analysis.

**Appendix G: Testis specific regulatory language**


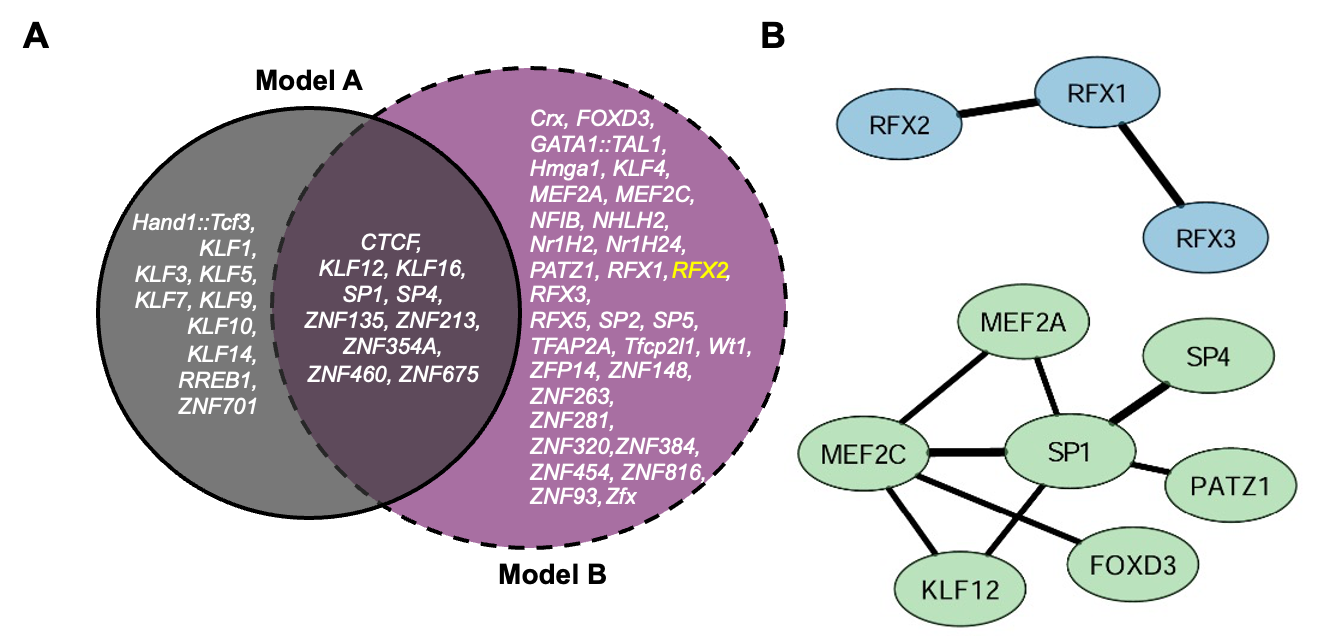
**Supplementary Figure 6:** (A) Overlap of top significant motifs in brain identified across Models A and B found among JASPAR hits. TSProm was applied to testis data to reveal transcription factors uniquely enriched in testis-specific promoters. (B) Biclustering results for 2 informative clusters where Blue cluster is *Fork_Zn* cluster and green is *Fork_MADS_Zn.*

**Supplementary Table 7:** Bi-clustering results of motifs found across different testis specific transcripts based on presence of absence in a 3k long sequence for Model B. Best bi-clustering is for 9 clusters with a silhouette score of 0.803.

| **Cluster** | **# TSS** | **Motif examples** | **Type** | **Key TFs** | **Dominant TF Families** |
| --- | --- | --- | --- | --- | --- |
| Nuc_ZnF | 2971 | CGAGGTCAGGAGT;  TTTTTTTTTTTTTT | Major | Nr1H2, Nr1H4, ZNF384 | C2H2 ZnF; Nuclear receptor (C4 ZnF) |
| Fork_ZnF | 149 | CCTGCCTGGCCCGC; GTGGCCAGGGTAAGG | Major | PRDM9, RFX1–5, ZNF213 | C2H2 ZnF; Forkhead/winged-helix |
| Tata_ZnF | 953 | ACTAAAAATAAAAAA; CTCAAATAAAAAAAAA | Major | Hmga1, ZNF384 | C2H2 ZF; TATA-binding proteins |
| Fork_MADS_ZnF | 1377 | CCCAGCAGGGCAGGACAGAG; GGCGGCGGCGGCGGG | Major | CTCF, FOXD3, GATA1::TAL1, MEF2A/C, SP1–5 | C2H2 ZF; Forkhead; MADS; bHLH |
| Homeo | 2150 | GCCCCCGCCTCCCGC; GCCTGTAATCCCAG | Major | CTCF, Crx, PRDM9, SP1–2 | C2H2 ZnF; Homeodomain |
| bHLH | 56 | CCTCAGTCGCAGCCCCGTCC; TCTTTAATAAAAAA | Minor | CTCF, NHLH2, PRDM9, ZNF354A | bHLH; C2H2 ZnF |
| bHSH | 29 | GGCGGGGGGGGCGGGTGGGG | Minor | CTCF, KLF4, TFAP2A, PRDM9 | bHSH; C2H2 ZF |
| SMAD | 3 | GCCGGGGGAAAATGGGGGGG | Minor | CTCF, NFIB, PRDM9 | C2H2 ZF; SMAD/NF-1 domain |
| Zn | 2 | CCGGCCTCGGCCTCCTCCGC | Minor | CTCF, PATZ1, ZNF454, ZNF460 | C2H2 ZF |

**Appendix H: High-Attention Regions Reveal Significant Global TSp Regulatory Motifs**

We summarize the significant top motifs identified from the JASPAR database among the significant motifs identified in all TSp promoters in human. We identify rich ‘CG’ repeat.

**Supplementary Table 8:** Representative significant motifs identified from enrichment analysis from the JASPAR database [13].

| **Query ID** | **Target ID** | **p-value** | **q-value** | **Overlap** | **Query consensus** | **Target consensus** | **Strand** | **TF name** | **Class** | **Family** |
| --- | --- | --- | --- | --- | --- | --- | --- | --- | --- | --- |
| TGCAGCTGTG | MA0521.3 | 4.50E-07 | 0.00052 | 6 | TGCAGCTGTG | CAGCTG | + | Tcf12 | bHLH | E2A |
| TGCAGCTGTG | MA0633.3 | 6.24E-06 | 0.00206 | 6 | TGCAGCTGTG | CAGCTG | + | Twist2 | bHLH | Tal-related |
| TGCAGCTGTG | MA1993.2 | 6.24E-06 | 0.00206 | 6 | TGCAGCTGTG | CAGCTG | + | Neurod2 | bHLH | Tal-related |
| TGCAGCTGTG | MA1997.2 | 6.24E-06 | 0.00206 | 6 | TGCAGCTGTG | CAGCTG | + | Olig2 | bHLH | Tal-related |
| GGAGGCYGAGGYDGS | MA1596.1 | 1.71E-06 | 0.00456 | 12 | GGAGGCCGAGGTGGG | CTCGGGAGGCTGAGGC | – | ZNF460 | C2H2 | Zinc fingers |
| GGGAGGCWGAA | MA1965.2 | 1.19E-06 | 0.00559 | 6 | GGGAGGCAGAA | GGGAGG | – | SP5 | C2H2 | Krüppel-related |
| GRTGRGGRAAAGGRGGVWGG | MA1723.2 | 1.21E-06 | 0.00566 | 18 | GATGAGGGAAAGGAGGAAGG | GGTGGGCAGGGAGGAAGCAG | + | PRDM9 | C2H2 | Multiple ZFs |
| TGCAGCTGTG | MA1635.2 | 2.17E-05 | 0.00626 | 6 | TGCAGCTGTG | CAGCTG | + | BHLHE22 | bHLH | Tal-related |
| CCGGGAGGYGGAG | MA1596.1 | 1.71E-06 | 0.00801 | 13 | CCGGGAGGCGGAG | CTCGGGAGGCTGAGGC | – | ZNF460 | C2H2 | Zinc fingers |
| GGGGGCCC | MA1548.2 | 4.98E-06 | 0.01154 | 8 | GGGGGCCC | GGGGGCCC | – | PLAGL2 | C2H2 | Zinc fingers |
| TGCAGCTGTG | MA1641.2 | 6.22E-05 | 0.01487 | 8 | TGCAGCTGTG | ACAGCTGT | + | MYF5 | bHLH | MyoD/ASC |
| TGCAGCTGTG | MA1472.3 | 6.76E-05 | 0.01487 | 8 | TGCAGCTGTG | ACAGCTGT | + | Bhlha15 | bHLH | Tal-related |
| TGCAGCTGTG | MA0048.3 | 6.39E-05 | 0.01487 | 9 | TGCAGCTGTG | GCAGCTGCG | – | NHLH1 | bHLH | Tal-related |
| TGCAGCTGTG | MA0500.3 | 8.00E-05 | 0.01546 | 8 | TGCAGCTGTG | GCAGCTGC | + | MYOG | bHLH | MyoD/ASC |
| DYYYDGYTAAWWTWTKTTTT | MA1978.2 | 1.42E-05 | 0.01659 | 20 | TTCTAGCTAATTTTTTTTTT | AATTAGTCCATTTATATTTA | – | ZNF354A | C2H2 | Zinc fingers |
| WRGGAGGSGMYGGGCYGG | MA1522.2 | 3.94E-06 | 0.01846 | 8 | AAGGAGGGGATGGGCTGG | GGGAGGGG | – | MAZ | C2H2 | Zinc fingers |
| TGCAGCTGTG | MA1619.2 | 1.25E-04 | 0.02219 | 8 | TGCAGCTGTG | ACAGCTGT | + | Ptf1A | bHLH | Tal-related |
| GGGAGGCWGAA | MA1596.1 | 9.49E-06 | 0.02228 | 11 | GGGAGGCAGAA | CTCGGGAGGCTGAGGC | – | ZNF460 | C2H2 | Zinc fingers |
| CCGGGAGGYGGAG | MA1587.1 | 1.06E-05 | 0.02490 | 13 | CCGGGAGGCGGAG | TCAGGAGGTCGAGG | – | ZNF135 | C2H2 | Zinc fingers |
| WCCAGCCTGGGCRAC | MA2121.1 | 5.69E-06 | 0.02671 | 12 | TCCAGCCTGGGCAAC | CGCAGCCTGGGC | – | ZNF213 | C2H2 | Zinc fingers |
| AGCTAGACCTCCCTGCTGC | MA1628.2 | 6.13E-06 | 0.02874 | 7 | AGCTAGACCTCCCTGCTGC | CCTGCTG | – | Zic1::Zic2 | C2H2 | Zinc fingers |
| AGCTAGACCTCCCTGCTGC | MA0697.3 | 1.23E-05 | 0.02884 | 7 | AGCTAGACCTCCCTGCTGC | CCTGCTG | – | Zic3 | C2H2 | Zinc fingers |
| GGAGGCYGAGGYDGS | MA1972.1 | 8.56E-05 | 0.03985 | 15 | GGAGGCCGAGGTGGG | GGAGGCACTGGAATG | + | ZFP14 | C2H2 | Zinc fingers |
| GGAGGCYGAGGYDGS | MA1723.2 | 7.91E-05 | 0.03985 | 15 | GGAGGCCGAGGTGGG | GGTGGGCAGGGAGGAAGCAG | + | PRDM9 | C2H2 | Zinc fingers |
| TTTTTKTWTTTTTWK | MA1125.2 | 6.49E-05 | 0.04132 | 8 | TTTTTTTTTTTTTTG | TTTTTTTT | – | ZNF384 | C2H2 | Zinc fingers |
| GGAGGCYGAGGYDGS | MA1587.1 | 9.98E-05 | 0.04223 | 11 | GGAGGCCGAGGTGGG | TCAGGAGGTCGAGG | – | ZNF135 | C2H2 | Zinc fingers |
| TGCAGCTGTG | MA0499.3 | 2.89E-04 | 0.04456 | 9 | TGCAGCTGTG | GCACCTGTC | + | MYOD1 | bHLH | MyoD/ASC |
| CAGCWMYTYGGGAGG | MA1965.2 | 9.80E-06 | 0.04599 | 6 | CAGCTACTTGGGAGG | GGGAGG | – | SP5 | C2H2 | Krüppel-related |

**References**

1. Georgakopoulos-Soares, I., et al., *Transcription factor binding site orientation and order are major drivers of gene regulatory activity.* Nature communications, 2023. **14**(1): p. 2333.

2. Tharakaraman, K., et al., *The biological function of some human transcription factor binding motifs varies with position relative to the transcription start site.* Nucleic acids research, 2008. **36**(8): p. 2777–2786.

3. Veerla, S. and M. Höglund, *Analysis of promoter regions of co-expressed genes identified by microarray analysis.* BMC bioinformatics, 2006. **7**(1): p. 384.

4. Zhang, P., H. Zhang, and H. Wu, *iPro-WAEL: a comprehensive and robust framework for identifying promoters in multiple species.* Nucleic Acids Research, 2022. **50**(18): p. 10278–10289.

5. Zhang, P. and H. Wu, *IChrom-deep: an attention-based deep learning model for identifying chromatin interactions.* IEEE Journal of Biomedical and Health Informatics, 2023. **27**(9): p. 4559–4568.

6. Vavouri, T. and B. Lehner, *Human genes with CpG island promoters have a distinct transcription-associated chromatin organization.* Genome biology, 2012. **13**(11): p. R110.

7. Landolin, J.M., et al., *Sequence features that drive human promoter function and tissue specificity.* Genome research, 2010. **20**(7): p. 890–898.

8. Saxonov, S., P. Berg, and D.L. Brutlag, *A genome-wide analysis of CpG dinucleotides in the human genome distinguishes two distinct classes of promoters.* Proceedings of the National Academy of Sciences, 2006. **103**(5): p. 1412–1417.

9. Smedley, D., et al., *BioMart–biological queries made easy.* BMC genomics, 2009. **10**(1): p. 22.

10. Feng, C., et al., *KnockTF 2.0: a comprehensive gene expression profile database with knockdown/knockout of transcription (co-) factors in multiple species.* Nucleic Acids Research, 2024. **52**(D1): p. D183–D193.

11. Xu, M., et al., *TF-Marker: a comprehensive manually curated database for transcription factors and related markers in specific cell and tissue types in human.* Nucleic acids research, 2022. **50**(D1): p. D402–D412.

12. Grant, C.E. and T.L. Bailey, *XSTREME: Comprehensive motif analysis of biological sequence datasets.* BioRxiv, 2021: p. 2021.09. 02.458722.

13. Rauluseviciute, I., et al., *JASPAR 2024: 20th anniversary of the open-access database of transcription factor binding profiles.* Nucleic acids research, 2024. **52**(D1): p. D174–D182.
